# The Association of Pain Levels and Low Physical Activity among Older Women

**DOI:** 10.3390/geriatrics6040103

**Published:** 2021-10-23

**Authors:** Thelma J. Mielenz, Jing Tian, Kevin D. Silverman, Adam M. Whalen, Sneha Kannoth, Laura L. Durbin, Alexander S. Perlmutter, Qian-Li Xue

**Affiliations:** 1 Department of Epidemiology, Columbia University Mailman School of Public Health, 722 West 168th Street, New York, NY 10032, USA; kdsilverman19@gmail.com (K.D.S.); amw2275@cumc.columbia.edu (A.M.W.); sk4690@cumc.columbia.edu (S.K.); asp2183@cumc.columbia.edu (A.S.P.); 2 Department of Biostatistics, Bloomberg School of Public Health, Johns Hopkins University, 615 North Wolfe Street, Baltimore, MD 21205, USA; jtian4@jhmi.edu; 3 Lenox Hill Hospital, Department of Medicine, Northwell Health, 100 East 77th Street, New York, NY 10075, USA; ldurbin@northwell.edu; 4 Division of Geriatric Medicine and Gerontology, School of Medicine, Johns Hopkins University, 733 North Broadway, Baltimore, MD 21205, USA; qxue@jhmi.edu

**Keywords:** aging, women’s health, chronic pain, physical inactivity

## Abstract

There is an integral research gap regarding whether there is a relationship between pain levels and low physical activity among older women. This is a secondary analysis of a longitudinal cohort study, the Women’s Health and Aging Study (WHAS) II. Our analyses included 436 community-dwelling women between the ages of 70 and 79, who were followed for 10.5 years. We employed marginal structural modeling, which controls for time-dependent confounding, with the aim of assessing the potential direct association between pain levels and low physical activity and assess a graded relationship. Compared to women with no pain, those with widespread pain were nearly half as likely to be moderately active versus low active (aOR: 0.46, 95% confidence interval (CI): 0.22, 0.96). A graded association was observed across the four pain levels (no pain or mild pain, other pain, moderate or severe lower extremity pain, and widespread pain) on low physical activity. Our findings indicate that reducing chronic widespread pain in older women may increase moderate physical activity, and therefore reduce the downstream health risks of low physical activity, including morbidity and mortality risk.

## 1. Introduction

Chronic widespread pain is increasingly recognized as a public health challenge today. Widespread pain is defined as pain experienced in the upper extremities, lower extremities, and axial skeletal pain, with the individual rating their pain as at least moderate (≥4) on the 11-point numeric rating scale for at least one site [1]. A systematic review demonstrated that approximately one in ten adults in the general population report experiencing chronic widespread pain, and found that this experience is especially prevalent among women [2]. The treatment of chronic widespread pain is rather difficult, considering inadequate biomedical diagnoses, and recent concerns surrounding opioid therapies for chronic pain, including risk of opioid use disorder and overdose [3,4,5,6].

Due to the increased prevalence of chronic widespread pain among women and lack of adequate treatment, it is important to understand the scope of the problem and health outcomes that are associated with chronic widespread pain among women. Previous research examined the relationship between pain and low physical activity levels, using data from the Epidemiology of Functional Disorders Study (EPIFUND), a large prospective cohort (2182 participants, ages 25–65), and found that those with chronic widespread pain at baseline were more likely to report higher low physical activity levels at 32 months follow-up, compared to those with no pain [7]. There was also a graded relationship observed for physical activity levels across pain categories, including: no pain, some pain, and chronic widespread pain [7]. Similarly, using the National Health and Nutrition Examination Survey (NHANES), a large, nationally representative sample (3952 participants, aged 20+ years), researchers found an association between chronic widespread pain and low physical activity levels, as those with chronic widespread pain had reduced daily and moderate-to-vigorous physical activity levels (measured via accelerometry), compared to individuals with no chronic widespread pain [8].

Researchers have also examined a potential link between pain and disability among older women (65+ years), using the longitudinal Women’s Health and Aging Study (WHAS). Researchers found that the percentage of older women who developed a new disability over three years rose steadily in all pain categories (widespread pain; no pain or mild pain; moderate or severe lower extremity pain; and other pain), but this increase was most significantly pronounced among those who reported widespread pain [1]. It was concluded that the pathway from widespread pain to mobility difficulty was likely a direct relationship. Thus, regardless of other physical impairments, widespread pain may be a sufficient cause of mobility difficulty on its own. This laid the foundation for examining further negative impacts of chronic widespread pain among older adult women in WHAS II [1].

We aimed to determine whether levels of pain had a direct causal effect on physical activity levels in a cohort of women. We hypothesized that after adjustment of key time-dependent confounders, pain levels will have a direct association with low physical activity in WHAS II participants. A time-dependent confounder specifically identifies a covariate that is a risk factor for, or predictor of, the outcome of interest (e.g., low physical activity) and predicts subsequent exposure (e.g., pain) [9,10]. We are further interested in examining the relative contributions that four levels of pain have toward low physical activity over time, in assessing the potential graded relationship of increased pain contributing to increased low physical activity, after controlling for time-dependent confounding.

## 2. Materials and Methods

We followed the reporting standards from STROBE (Strengthening the Reporting of Observational Studies in Epidemiology) Statement [11].

### 2.1. Data Source and Study Sample

The Women’s Health and Aging Study (WAHS) II is a prospective cohort study of 436 community-dwelling women, who at baseline were 70–79 years old, cognitively fit (Mini Mental State Examination score greater than 24), and scored in the highest two thirds of their age group for physical functioning (defined as no difficulty on tasks or only difficulty in one out of four functional domains) [12,13,14]. This cohort is a companion cohort for the original WHAS study, which focused on the women scoring in the lowest one third for physical functioning in their community [12,15]. Women for both studies were recruited from Medicare beneficiaries in 12 connecting ZIP codes of Baltimore City and County, Maryland [16]. Baseline data were retrieved in 1994 and six follow-up physical examinations were conducted over 10.5 years, generally at 18-month intervals, excluding the 3-year interval between the second and the third follow-up examinations [16]. Out of the women who were screened and eligible for the study, there was a 49.5% response rate, with study participants reporting more diseases, higher education, and similar disability compared to those who did not participate [13].

### 2.2. Pain Level Assessment and Categorization

At baseline, study staff asked participants if they had experienced pain at several musculoskeletal joint sites (hands, wrists, hips, knees, feet, back, and chest) on most days for at least one month in the previous year. Severity of pain was determined by an 11-point numeric rating scale, where 0 indicated “no pain” and 10 indicated “severe or excruciating pain as bad as you can imagine”.1 Categorizations of pain location included: upper extremities (hand or wrist), lower extremities (hip, knee, or foot), and axial skeletal pain (back or chest). In accordance with prior research, pain levels were divided into four pain categories: widespread pain; moderate or severe lower extremity pain; other pain [not meeting the criteria for widespread or lower extremity pain, including mild pain (<4 on the numeric rating scale) in more than one site]; and no pain or mild pain (only mild pain in one site) [1].

### 2.3. Physical Activity

Study staff asked participants about their physical activity at baseline and at each follow-up physical examination visit. The questions included a subset of the Minnesota Leisure Time Activities Questionnaire [17]. Individuals were asked about whether they had participated, frequency of participation, and duration of participation (average minutes per session) for four activities: walking; dancing; bowling; and exercise (e.g., stretching, strengthening), along with two lifestyle activities: strenuous household chores (e.g., scrubbing, vacuuming); and strenuous outdoor chores (e.g., gardening). From this information, we calculated the kilocalorie expenditure per kilogram of body weight per day over a two-week period. We used the National Health Interview Survey’s cutoff points, creating three physical activity categories: low active (<1.5 kcal); moderately active (1.5 ≤ kcal < 3.0); and very active (≥3.0 kcal) [18].

### 2.4. Covariates

Our study included both time-dependent and time-independent covariates. Potential time-independent covariates included the following sociodemographic and health measures at baseline: age (number of years), race (white; black or other), education (number of years), comorbidities and health status, body mass index (BMI), smoking status (current; former; never), drinking status (usual drinker; not a usual drinker), pain medication use (yes; no), Geriatric Depression Scale (GDS) scores, and scores on the Short Physical Performance Battery (SPPB) [19,20,21]. Potential time-dependent covariates included the following: BMI, pain medication use, GDS scores, SPPB scores, and physical activity status at the previous visit.

In regards to further information pertaining to the measurement of the sociodemographic and health covariates, comorbidities were measured as a count variable of the number of adjudicated chronic conditions that an individual reported at baseline, including the following conditions: coronary artery disease, congestive heart failure, degenerative disc disease, spinal stenosis, hip fracture, osteoporosis, rheumatoid arthritis, stroke, Parkinson’s disease, pulmonary disease, diabetes mellitus, peripheral arterial disease, and cancer [14]. Individuals originally self-rated their health status on a four-item scale (excellent, good, fair, and poor) and the “excellent” and “good” answers were collapsed into the same category for the purpose of the analysis. BMI (weight (kg)/height^2^ (m^2^)) was broken into four categories according to well-established standards: <18.5 (underweight), 18.5–24.9 (normal weight), 25–29.9 (overweight), and >30 (obese).

The GDS is a frequently used 30-item depression scale, in which older adults provide simple yes/no answers to questions about depression and suicide ideation [16]. Previously, 14 or more positive responses has been used to indicate moderate to high levels of depressive symptoms [1]. The GDS has sound psychometric properties and is easy to administer with older adults [22,23].

The SPPB is a measure that assesses balance and physical functioning, with a specific focus on lower extremity function [20]. It includes three major components: standing balance (side-by-side, semi-tandem, and tandem positions), usual walking speed, and ability to rise from a chair (length of time used to rise five times with arms folded). A score of zero (unable to carry out the task) to four (best possible performance) is assigned to each of the tasks and summed to create a total SPPB score (highest possible score is 12). The SPPB is a reliable test that has been used in multiple population aging studies and has been shown to be predictive of hospitalizations, length of hospital stays, health outcomes after discharge from the hospital, and changes in functional and health status in older adults [20,24,25,26,27,28,29].

### 2.5. Statistical Analyses

The chi-squared test was employed to determine if pain categories and physical activity levels were independent from baseline characteristics. Fisher’s exact test was used only for categories where low counts were observed, while testing the independence of self-reported health and pain categories.

Marginal structural modeling was employed to estimate the causal association of the time-varying pain status with physical activity, controlling for time-dependent confounding over a 10.5-year period. The parameters of the model were estimated using an inverse-probability-of-treatment weighted estimator [10]. The following general steps were performed when fitting our marginal structural model: (1) define the study outcome and exposure; (2) consider all known or suspected confounders to approximate no unmeasured confounding; (3) define the treatment or exposure model to obtain the treatment weights; (4) define the censoring model to obtain the censoring weights; (5) calculate the inverse-probability-of-treatment weights; and (6) conduct a weighted analysis of outcome on exposure using standard software [10,30,31,32].

In our analysis, the exposure (or ‘treatment’) was time-varying pain status, and the outcome was physical activity status. We distinguished universal confounders that we must adjust for in the model (e.g., age) from ‘potential’ confounders and explored cross-sectional associations between potential confounders and pain, and between potential confounders and physical activity at each round. We retained potential confounders with *p*-values < 0.2 for most of the rounds. Based on this analysis, we chose the time-dependent and time-independent confounders to be used in our marginal structural modeling. Time-dependent confounders included BMI, pain medication use, GDS scores, SPPB scores, and previous visit physical activity status. Time-independent confounders included age, race, education, comorbidities and health status, smoking status, and drinking status [20].

At each round, we ran multinomial logistic regression models for pain (treated as a multinomial outcome), adjusting for our time-dependent and time-independent confounders. To better characterize the history of pain, we used the pain pattern by time *t* (whether the participant ever had widespread pain by time *t*; whether the participant ever had moderate or other pain, but not widespread pain by time *t*; whether the participant had no/mild pain all through time *t*) as the exposure in the model. We calculated the predicted probability of the observed pain pattern at each round for each study subject based on the model fitting and plotted the observed pain levels against the predicted levels to examine the experimental treatment assignment assumption. At each round, we also ran multinomial logistic regression models for pain (treated as a multinomial outcome) using only time-independent confounders, calculating the predicted probability of observed pain level at each round for each study subject, based on this model fitting. We calculated the subject-specific stabilized treatment weight as a ratio, with the denominator being the product of the round-specific weights from baseline to time *t*, using time-dependent and time-independent confounders, and the numerator being the product of the round-specific weights, using only time-independent confounders.

The next step was to define the censoring model to obtain the censoring weights to account for study attrition. We ran a logistic model for drop out (defined as those who had physical activity status missing at time t or not as the outcome), using both time-dependent and time-independent confounders. The predicted probability of the observed censoring became the denominator of the stabilized censoring weight. We then ran a logistic model for drop out (physical activity status missing at time *t* or not as the outcome), using only time-dependent confounders. The predicted probability of the observed censoring became the numerator of the stabilized censoring weight. The stabilized censoring weight was calculated by combining the aforementioned numerator and denominator into a ratio. We then calculated the stabilized final weight by multiplying the subject-specific stabilized treatment weight with the stabilized censoring weight. Finally, we ran a weighted analysis (using the stabilized final weight) of a pooled multinomial logistic regression of physical activity at time *t* with pain status at each visit and baseline confounders.

All analyses were conducted in Stata Version 11.0 (Stata, College Station, TX, USA) [33]. We used the cluster option in Stata to obtain robust estimates of standard errors and confidence intervals [33].

## 3. Results

From the baseline number of participants (*n* = 436) collected between 1994–1996, the follow-up visits two through seven had the following *n*’s: 418, 408, 335, 313, 285, and 153.

The average participant age was 73.9 years, and the mean educational attainment was 12.5 years of school (Table 1). The participants were predominantly white (81.1%). Few participants self-reported poor health (11%) or current smoking (10.2%), and some reported being usual drinkers (31.6%). The average comorbid disease count was 1.5 and the average GDS and SPPB scores (4.0 and 8.4, respectively) indicated that most women were not experiencing depressive symptoms and were not experiencing significant problems with physical functioning at baseline. BMI levels varied among participants, with 3.2% underweight, 35.7% normal weight, 37.8% overweight, and 23.3% obese. Only 13.1% reported having experienced widespread pain on most days for at least one month in the previous year, but 65.1% reported having ever used a pain medication.

Baseline characteristics that varied significantly (*p* < 0.05) by physical activity status included SPPB scores, GDS scores, disease count, self-reported health, and BMI (Table 2). Sedentary individuals had a lower SPPB score than those who were moderately or very active. There was a gradient observed with GDS scores and disease counts, with more active individuals reporting lower (better) GDS scores and lower disease counts. A majority of moderately and very active individuals reported being in excellent or good health, but the largest proportion of low active individuals (43.8%) reported being in fair health. Regarding BMI, 29.4% of low active individuals were classified as obese, compared to 19.2% of moderately active and 17.6% of very active individuals.

Among the participants who reported widespread pain, no baseline characteristics varied significantly by physical activity level. Table 3 reports the odds ratios from the marginal structural models for the association of pain status with low physical activity. Compared to women with no pain or mild pain, the odds ratio (OR) of women with widespread pain being moderately active (versus low active) was 0.46 (95% confidence interval (CI): 0.22, 0.96) and the OR of women with widespread pain being very active (versus low active) was 0.82 (95% CI: 0.45, 1.48). In other words, the likelihood of being moderately active (versus low active) is reduced by 54% among those with widespread pain compared to those with no pain or mild pain, and the likelihood of being very active (versus low active) is reduced by 18% (although not significant) among those with widespread pain compared to those with no pain or mild pain. Accordingly, the risk of being low active (versus very active) is similar for women who report widespread pain and women who report moderate or severe lower extremity pain, whereas the risk of being low active (versus moderately active) is approximately 84 percentage points greater among women who report widespread pain than among women who report moderate or severe lower extremity pain.

## 4. Discussion

Our findings indicate that women who reported widespread pain compared to women who reported no pain or mild are approximately twice as likely to be low active compared to being moderately active. Women who reported widespread pain compared to those who reported no pain or mild pain were almost 22% more likely to be low active compared to being very active, but this was not statistically significant. Interestingly, women with widespread and moderate or severe lower extremity pain compared to women with no pain or mild pain are only a quarter more likely to be low active compared to being moderately active. Nonetheless, the risk of being low active compared to being very active for women with moderate or severe lower extremity pain was found to be similar for women who reported no pain or mild pain.

One of our hypotheses was that widespread pain would have a direct causal association with low physical activity, adjusting for time-dependent confounders. Our results indicate that widespread pain can have a direct causal association with low physical activity. We further hypothesized that in older women, a graded relationship exists across the four included pain levels and low physical activity over time, when controlling for time-dependent confounding. We did find a graded trend across the four pain levels, but only chronic widespread pain was significant in the moderately active group. The very active group may increase widespread pain. Exploring this direct causal relationship of widespread pain to low physical activity is important because it is known that physical activity does not have to be vigorous to prevent the occurrence of these supplementary negative health outcomes [34].

If there is a direct causal association between widespread pain and low physical activity, it is important to consider the potential biological plausible mechanisms at work. The neuromatrix theory posits that pain is an output of a “widely distributed neural network” [35] (p. 38). According to this theory, pain causes the neuromatrix to adapt in negative ways. For instance, fibromyalgia (a syndrome characterized largely by widespread pain) may cause changes in the central nervous system at many different levels, which may result in maladaptive behaviors, such as reduced physical activity [35].

Another potential mechanism may be that individuals experiencing pain may catastrophize their symptoms, wherein pain is perceived as leading to negative consequences, resulting in fear avoidant behavior [7]. If pain is catastrophized, then individuals may be likely to avoid behaviors that they believe will increase their pain or any other perceived negative consequences [7]. Higher levels of catastrophizing are associated with persistence and increase in pain, which may be physiologically explained by an “amplification of cortical activation in relation to pain and/or dysregulation of endogenous opioids” [7] (p. 978).

Catastrophizing about pain could impact various bodily systems including the neuromuscular, cardiovascular, immune, and neuroendocrine systems, and may influence activity in the brain’s pain neuromatrix [36].

We suggest that intervening on chronic widespread pain could increase physical activity in older women, but the benefits may not end there. In addition to being associated with low physical activity, chronic widespread pain is associated with other negative health outcomes, including mental health outcomes such as depression and anxiety, and physical health outcomes such as obesity and frailty [35,37,38]. Furthermore, low physical activity itself is linked to physical health outcomes, such as obesity and frailty, and so, improvements across multiple related outcomes may be anticipated. Existing research that attempted to explain the mechanism behind the pain-obesity association did not find that characteristics, such as insulin resistance, inflammation, or painful comorbidities, could explain the relationship, and the authors suggested that an alternative pathway may include physical activity [37]. If our hypothesized causal pathway is correct, then intervening on chronic widespread pain could change not only low physical activity, but also prevent further downstream negative outcomes, such as early mortality [35]. In supporting this theory, researchers studied patients with fibromyalgia, and found that a 3-month multidisciplinary treatment program (including traditional pharmacological treatment, with physical and cognitive-behavioral therapy) led to enhancements across multiple related outcomes, including improved physical activity, exercise regularity, and functional status [39].

Our study notes several limitations. Unmeasured confounding is an issue for marginal structural modeling, and there is potential for unmeasured confounding in our study as we did not include variables of catastrophizing or pain avoidance, as well as external factors such as area level deprivation. Physical activity was self-reported in this study, and such subjective measures of physical activity have been shown to be less valid, accurate, and reliable than objective measures (e.g., accelerometry) [40]. Our pain measurement was limited to location and severity, and did not incorporate other important aspects of pain such as behavior and interference. Furthermore, only 13.1% of our sample reported experiencing widespread pain, making for limited cell counts on this exposure level.

Our research provides many implications for new research, in that future studies should confirm these findings using objective assessments of physical activity and by including more comprehensive measurements of pain with more pain domains. These studies should also consider oversampling on individuals with widespread pain and should include more measured confounders. We further recommend that future studies employ marginal structural modeling to avoid biased estimates.

## Figures and Tables

**Table 1:** Baseline Characteristics in Women’s Health and Aging Study II, Baltimore, Maryland, 1994–2009 (*N* = 436).

Age, Mean Years (SD)	73.9 (2.80)
Education, Mean Years (SD)	12.5 (3.3)
SPPB Score, Mean (SD)	8.4 (2.1)
GDS, Mean (SD)	4.0 (3.8)
Diseases, Mean (SD)	1.5 (1.0)
Health, No. (%)	
Excellent/good	214 (49.1)
Fair	174 (39.9)
Poor	48 (11.0)
Race, No. (%)	
White	353 (81.1)
Black and other	82 (18.9)
BMI ^a^, No. (%)	
<18.5 (Underweight)	14 (3.2)
18.5–24.9 (Normal weight)	155 (35.7)
25–29.9 (Overweight)	164 (37.8)
>30 (Obese)	101 (23.3)
Smoking Status, No. (%)	
Never Smoker	237 (54.7)
Former Smoker	152 (35.1)
Current Smoker	44 (10.2)
Alcohol Consumption, No. (%)	
Usual Alcohol Drinker	137 (31.6)
Does Not Drink Alcohol Usually	296 (68.4)
Ever Used Pain Medication, No. (%)	284 (65.1)
Ever Reported Widespread Pain, No. (%)	57 (13.1)

Abbreviations: BMI, Body mass index; GDS, Geriatric Depression Scale; SPPB, Short Physical Performance Battery. ^a^ Weight (kg)/height^2^ (m^2^).

**Table 2:** Baseline Characteristics and Physical Activity Level in Women’s Health and Aging Study II, Baltimore, Maryland, 1994–2009 (*N* = 433) ^a^.

Characteristic	Physical Activity Level			
	Low Active ^b^ (*N* = 185)	Moderately Active ^c^ (*N* = 99)	Very Active ^d^ (*N* = 149)	*p* Value
Age, Mean Years (SD)	74.2 (2.7)	73.3 (2.8)	74 (2.9)	0.05
Education, Mean Years (SD)	12.2 (3.3)	12.7 (3.0)	12.7 (3.5)	0.31
SPPB Score, Mean (SD)	8 (2.2)	8.6 (2.0)	8.7 (1.9)	0.001
GDS, Mean (SD)	4.8 (4.1)	3.9 (3.6)	3.3 (3.4)	0.001
Diseases, Mean (SD)	1.7 (1.1)	1.5 (1.1)	1.3 (0.9)	0.004
Health, No. (%)				0.001
Excellent and Good	74 (40.0)	52 (52.5)	88 (59.1)	
Fair	81 (43.8)	38 (38.4)	54 (36.2)	
Poor	30 (16.2)	9 (9.1)	7 (4.7)	
Race, No. (%)				0.47
White	146 (79.4)	80 (80.8)	126 (84.6)	
Black and Other	38 (20.7)	19 (19.2)	23 (15.4)	
BMI ^e^, No. (%)				0.02
<18.5 (Underweight)	2 (1.1)	6 (6.1)	6 (4.1)	
18.5–24.9 (Normal weight)	69 (37.5)	32 (32.3)	53 (35.8)	
25–29.9 (Overweight)	59 (32.1)	42 (42.4)	63 (42.6)	
>30 (Obese)	54 (29.4)	19 (19.2)	26 (17.6)	
Smoking Status, No. (%)				0.70
Never Smoker	96 (51.9)	59 (59.0)	82 (55.0)	
Former Smoker	67 (36.2)	31 (31.3)	54 (36.2)	
Current Smoker	22 (11.9)	9 (9.1)	13 (8.7)	
Alcohol Consumption, No. (%)				0.22
Usual Alcohol Drinker	52 (28.1)	30 (30.3)	55 (36.9)	
Does Not Drink Alcohol Usually	133 (71.9)	69 (69.7)	94 (63.1)	
Current Pain Medication Use, No. (%)	55 (29.7)	23 (23.2)	43 (28.9)	0.48
Ever Used Pain Medication, No. (%)	112 (60.5)	68 (68.7)	103 (69.1)	0.19

^a^ Three participants out of the original 436 were missing physical activity data. ^b^ <1.5 kilocalories expended per kilogram of body weight per day over a two-week period. ^c^ 1.5. ≤ kilocalories expended per kilogram of body weight per day over a two-week period < 3.0. ^d^ ≥3.0 kilocalories expended per kilogram of body weight per day over a two-week period. ^e^ Weight (kg)/height^2^ (m^2^).

**Table 3:** Odds Ratios from Marginal Structural Models for the Association of Pain Status with Low Physical Activity, Women’s Health and Aging Study II, Baltimore, MD, USA, 1994–2009.

Characteristic	Odds Ratio ^b^	95% Confidence Interval
Low Active	1.00	
Moderately Active ^a^		
Widespread Pain	0.46	0.22, 0.96
Moderate or Severe Lower Extremity Pain	0.75	0.52, 1.06
Other Pain	1.03	0.67, 1.59
Very Active ^a^		
Widespread Pain	0.82	0.45, 1.48
Moderate or Severe Lower Extremity Pain	0.83	0.59, 1.15
Other Pain	1.04	0.70, 1.56

^a^ Reference category is no pain or mild pain. ^b^ Model adjusted by baseline age, race, education, diseases, health, and clustering by participants.

## Data Availability

WHAS II uses publicly available data.
